# Prevalence of the metabolic syndrome using two proposed definitions in a Japanese-Brazilians community

**DOI:** 10.1186/1758-5996-4-38

**Published:** 2012-08-18

**Authors:** Maria C Foss-Freitas, Patricia M Gomes, Regina CG Andrade, Roberta C Figueiredo, Ana E Pace, Amaury L Dal Fabbro, Luciana Z Monteiro, Laercio J Franco, Milton C Foss

**Affiliations:** 1 Department of Internal Medicine, Sao Paulo University-Brazil, Sao Paulo, Brazil; 2 Department of Social Medicine, Sao Paulo University-Brazil, Sao Paulo, Brazil; 3 Ribeirão Preto Medical School, Ribeirão Preto Pharmacy School, Sao Paulo University-Brazil, Sao Paulo, Brazil; 4 Ribeirão Preto Nurse School, Sao Paulo University-Brazil, Sao Paulo, Brazil

**Keywords:** Metabolic syndrome, Japanese-Brazilians, IDF, NCEP, Waist circumference

## Abstract

Metabolic Syndrome (MetS) is associated with increased risk of morbi-mortality, thus the characterization of the population magnitude of this syndrome is critical for allocating health care. However, prevalence estimates of MetS in the same population could differ depending on the definition used. Therefore, we compared the prevalence of the MetS using definitions proposed by: National Cholesterol Education Panel Revised (NCEP) and International Diabetes Federation (IDF) 2009 in a Japanese-Brazilians community (131 individuals, age 57 ± 16 years, 1st and 2nd generation). All individuals went through a clinical and laboratorial evaluation for assessment of weigh, height, waist circumference, blood pressure, triglycerides, HDL-cholesterol and fasting plasma glucose. The prevalence of MetS was 26.7% (n = 35) and 37.4% (n = 49) under the NCEP and IDF definitions, respectively. Despite higher blood pressure measurements, waist circumference and serum triglyceride levels and lower HDL cholesterol levels (p < 0.01), individuals identified with MetS did not show increased blood glucose levels. IDF definition classified 14 individuals (10.7%) with MetS that were not classified under the NCEP and 35 individuals were identified with MetS by both criteria. We observed, in this group, more severe lipid disorders, compared to individuals identified only under the IDF definition, and the BMI and waist circumference (p = 0.01; p = 0.006, respectively) were lower. In conclusion, the IDF revised criteria, probably because of the ethnic specific values of waist circumference, was able to identify a larger number of individuals with MetS. However, our data suggesting that additional studies are necessary to define best MetS diagnostic criteria in this population.

## Introduction

The metabolic syndrome (MetS) is an aggregation of biochemical and physical conditions that presage the development of atherosclerotic cardiovascular disease. Although it has been known for more than 80 years, the clustering received scant attention until 1988 when Reaven described syndrome X [1]. Further, other metabolic abnormalities have been considered as part of the syndrome, like abnormal weight or weight distribution, inflammation, microalbuminuria, hyperuricemia, and abnormalities of fibrinolysis and coagulation [2]. The constellation of metabolic abnormalities of the MetS includes glucose intolerance, insulin resistance, central obesity, dyslipidaemia and hypertension, all well documented risk factors for cardiovascular disease.

People with the MetS are at increased risk of all-cause and cardiovascular disease (CVD) mortality [3-5]. In addition, components of the MetS are risk factors for diabetes [6]. Over the last decade several clinical criteria for MetS have been developed [7,8]. Clinically defined MetS criteria were proposed by the National Cholesterol Education Program (NCEP) (The Expert Panel on Detection, Evaluation, and Treatment of High Blood Cholesterol in Adults: Executive summary of the third report of the National Cholesterol Education Program (NCEP) Expert Panel on Detection, Evaluation, and Treatment of High Blood Cholesterol in Adults, 2001) [9] and were subsequently revised (NCEP-R) by lowering the threshold for blood glucose to correspond to the impaired fasting glucose cutoff of the American Diabetes Association [10]. Most recently, the International Diabetes Federation (IDF) proposed consensus criteria for identification of MetS [11].

MetS is associated with increased risk of cardiovascular morbi-mortality, thus understanding the dimension of this syndrome is critical for allocating health care and research resources. However, by the use of different definitions, prevalence estimates of MetS in the same population could differ depending on the definition used. In Mumbuca/Guatapara, located in Sao Paulo state, Brazil, there is a population that migrated from Japan in 1962, that lives as a semi-rural community and has retained much of their traditional habits. Thus the propose of this study was to compare the prevalence of the MetS using definitions proposed by: NCEP and IDF definitions in a Japanese-Brazilians community.

## Subjects and methods

This study was a cross-sectional evaluation of a Japanese-Brazilians Community in Mumbuca/Guatapara, Sao Paulo State. We evaluated 131 individuals from first and second generations, what represents 66.8% of the adults older than 20 years of this community. Individuals not Japanese descendent or younger than 20 years, were not included in the study. The patients comprised 54 (41.2%) men and 77 (58.8%) women with a mean age of 57 ± 16 years. Clinical and laboratorial evaluations were performed by trained professionals with adequate equipment. The Ethics Committee of the University Hospital of the School of Medicine of Ribeirão Preto-USP approved the study protocol and all participants gave written informed consent to participate in the study.

### Clinical examination

All participants underwent a clinical examination after fasting for at least 12 h. Anthropometric evaluation was performed with the participant barefoot and wearing light clothing. The weight in kilogram was assessed in an electronic scale (Filizola®) and the height, in centimeters, was measured with a portable stadiometer. The body mass index (BMI) was calculated by dividing weight (kg) by height (m) squared. Patients were categorized according to BMI values advocated by the International Obesity Task Force (IOTF) for the Asian population: between 23 and 24.9 kg/m^2^ for overweight and ≥ 25 kg/m^2^ for obesity [12].

Waist circumference was measured midway between the lower edge of the last rib and the iliac crest, to access the abdominal obesity (AO). To check the blood pressure (BP), we used an automated device (OMRON®, Itapevi, SP Brazil) with appropriate cuff. The normal values of BP were those recommended by the VI Brazilian Guidelines for Hypertension (2010) [13], and was considered the final blood pressure, the mean of the last two of three consecutive measurements. All instruments were checked for calibration before using.

A fasting blood sample was obtained, and concentrations of triglycerides, HDL cholesterol, basal insulin and glucose were assayed using automated techniques. For measures of triglycerides (TG) and HDL-cholesterol (HDL-C) we used an automated spectrophotometric method (Wiener lab, Rosario, Argentina). Glucose was measured by enzymatic method (hexokinase).

### Metabolic syndrome

According to NCEP definition, MetS is present if three or more of the following are present: high waist circumference (> 102 cm for males and > 88 cm for females), high triglyceride levels (≥ 150 mg/dL), low HDL cholesterol levels (< 40 mg/dL for men and < 50 mg/dL for women), elevated blood pressure (≥ 130/85 mmHg) and elevated glucose values (≥ 100 mg/dL). According to IDF definition, MetS is present if there is present three or more of the following: high waist circumference (≥ 90 cm for men and ≥ 80 cm for women, in the Japanese group), high triglyceride level, low HDL cholesterol level, elevated blood pressure and elevated glucose value. In addition to the clinical criteria, individuals who reported use of antihypertensive or antidiabetic agents were classified as having elevated blood pressure and glucose, respectively, for both MetS definitions.

### Statistical analysis

According to the NCEP and IDF definitions the individuals studied were divided in two groups. Individuals identified by both definitions were classified as concordant group and individuals identified with MetS only by the IDF definition were classified as discordant group. After testing for normal distribution, an ANOVA statistic test was used to analyze the relation between each variable involved in the MetS classification according to the two different definitions used. The Kappa Coefficient of Agreement was determined to evaluate the concordance between the two definitions. A significance level of 0.05 (α = 5%) was adopted, and levels below this were considered significant. All analyses were conducted using SAS software, version 9.1 (SAS Institute, Cary, NC).

## Results

**Figure 1:**
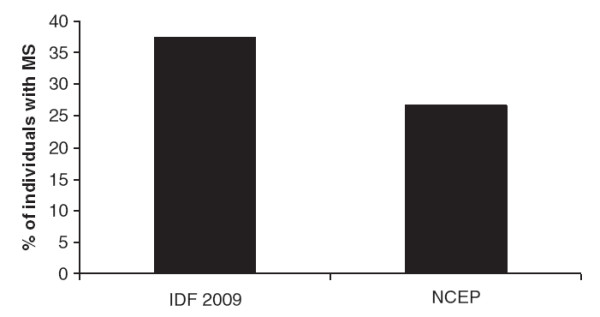
Prevalence (%) of individuals with metabolic syndrome according to the IDF 2009 and NCEP criteria.

Analyzing the 131 patients we observed that 37.4% (n = 49) of the individuals studied were classified as having MetS. Although, the prevalence of MetS was 26.7% (n = 35) and 37.4% (n = 49) under the NCEP and IDF definitions, respectively (Figure 1). Individuals identified with MetS showed higher blood pressure measurements, waist circumference, elevated serum triglyceride levels and lower HDL cholesterol levels (p < 0.01, Table 1), as expected, however they did not show significant higher fasting plasma glucose levels. All individuals identified under the NCEP definitions were also identified under the IDF. The two definitions showed a high concordance according to the Kappa coefficient (0.76, confidence interval: 0.64; 0.87).

**Table 1:** Clinical and laboratory characteristics of the population according to the four different groups

	**Fasting plasma glucose (mg/dL)**	**HDL cholesterol (mg/dL)**	**Triglyceride (mg/dL)**	**BMI (kg/m**^**2**^**)**	**Waist circumference (cm)**	**Sistolic blood pressure (mmHg)**	**Diastolic blood pressure (mmHg)**
**Total**	92.6 ± 19.5	49.4 ± 13.2	145.1 ± 87.0	24.7 ± 4.0	84.4 ± 10.5	135.0 ± 25.5	79.0 ± 12.6
**NCEP**	99.6 ± 15.3	41.2 ± 9.8	212.6 ± 80.1	27.3 ± 4.5	93.0 ± 11.7	149.8 ± 29.6	85.2 ± 15.3
**IDF**	97,5 ± 14.6	42.9 ± 9.4	188.4 ± 81.0**	26.8 ± 4.27*	91.5 ± 10.7*	148.3 ± 27.3	85.2 ± 13.5
**Discordant**	91.8 ± 11.8^#^	47.7 ± 6.5^#^	119.8 ± 32.8^#^	25.1 ± 2.93^#^	87.5 ± 6.8^#^	146.2 ± 21.3	85.4 ± 8.5
**Concordant**	106.0 ± 19.5	43.0 ± 10.6	213.9 ± 83.4	22.5 ± 2.96	78.9 ± 6.5	138.2 ± 9.7	80.7 ± 15.6

*p < 0.05 comparing IDF and group Concordant; **p < 0.05 comparing IDF and group Discordant ^#^p < 0.05 comparing group Concordant and group Discordant.

Among all participants, IDF definition classified 14 (10.7%) individuals with MetS that were not classified under the NCEP (discordant) and 35 individuals were identified with MetS with both criteria (concordant). We observed, in the last group, higher triglycerides and lower HDL (p < 0.01) and higher fasting plasma glucose (p = 0.05) compared to individuals identified only under the IDF definition. Interestingly the BMI and waist circumference (p = 0.01; p = 0.006, respectively) were lower in the concordant compared to discordant groups. However, there was no difference among the groups according to age, total cholesterol, LDL cholesterol, and blood pressure levels.

Individuals identified with MetS were mostly female (36.3%). In addition, individuals with MetS independent of the diagnostic criteria, showed increased levels of triglycerides compared to the discordant group (p = 0.004) and increased values of BMI (p = 0.02) and waist circumference (p = 0.007) compared to the concordant group.

## Discussion

In this study we have provided the first assessment of the prevalence of MetS, in the Japanese-Brazilian community of Mumbuca/Guatapara, using the recently revised IDF criteria [11]. We also compared it to the prevalence using the more traditional NCEP definition. Besides, both IDF and NCEP focus specifically on waist circumference, which is a surrogate measure of central obesity, the IDF definition builds upon the NCEP criteria, but differs in two key aspects. First, the IDF has lowered the threshold for waist circumference from 102 to 94 cm. Second, waist circumference is different according to ethnical characteristics. Previous studies have shown that using a lower waist circumference threshold within the context of MetS increases the prevalence, but decreases the risk of mortality [3] and type 2 diabetes [14].

Previous studies have observed an increased prevalence of MetS among American-Japanese compared to native Japanese, demonstrating the influence of the westernization of the lifestyle [15]. Data like this has raised interest in assessing the prevalence of MetS in Japanese migrant populations. Mumbuca/Guatapara, located in Sao Paulo state, Brazil, becomes an interesting population to study the effect of the western lifestyle in Japanese communities, since they represents the last Japanese migration to Brazil and living as a semi-rural community they retained much of their traditional habits.

Several studies have reported the difference in MetS prevalence estimates depending on the definition used [16-18]. However, there is a tendency to define diagnostic criteria based on ethnicity and central obesity [19]. The American Heart Association/National Heart, Lung, and Blood Institute, had, already, recommended different values to Asian population [10], and in a recent study in a Japanese-Brazilian community it was observed that the MetS prevalence was higher using the NCEP criteria for Asians [20]. We believe that our data reinforce this idea, since the IDF criteria showed the higher MetS prevalence compared to the NCEP definition.

In addition, we demonstrate that individuals with MetS had significant lipid disorders, higher prevalence of central obesity and elevated blood pressure levels, which are well known cardiovascular disease risk factors, but not higher fasting plasma glucose levels. Interestingly, concordant group, composed by those identified with MetS by both criteria, did present higher glucose and lipid levels, but lower BMI and waist circumference values. Therefore, our data highlight the importance of insulin resistance in the etiology of MetS in Japanese-Brazilians [21]. The World Health Organization (WHO) and European Group for the study of Insulin Resistance (EGIR) had proposed diagnostic criteria based on the detection of insulin resistance. In fact, it had been demonstrated that the WHO criteria detects more individuals with MetS, having more glucose metabolism and lipid disorders, compared to NCEP criteria [22]. Besides, the same study, recommends the use of NCEP criteria with reduced waist cutoff for the MetS detection, based in its practicability.

In conclusion, the high prevalence of MetS shows that Japanese-Brazilian community of Mumbuca is in increased risk for cardiovascular disease. According to the increased deterioration of the glucose and lipid metabolism, associated with central obesity, individuals identified with MetS would benefit most from lifestyle adjustment. The IDF revised criteria, because of the ethnic specific values of waist circumference, was able to identify a larger number of individuals with MetS.

## Competing interests

The authors declare that they have no competing interests.

## Authors’ contributions

MCFF, LJF and MCF participated in the design of the study. MCFF, RCGA, RCF, AEP, ALDF, LJF and MCF performed the data collection. LZM performed the statistical analysis. MCFF, PMG and MCF wrote the paper. All authors read and approved the final manuscript.
